# Toxoplasma rhoptry proteins that affect encephalitis outcome

**DOI:** 10.1038/s41420-023-01742-1

**Published:** 2023-12-04

**Authors:** Xinlei Wang, Lai Qu, Jie Chen, Yufen Jin, Kaisong Hu, Zhengjie Zhou, Jiaqi Zhang, Yiming An, Jingtong Zheng

**Affiliations:** 1 Department of Clinical Laboratory, The Second Hospital of Jilin University, Changchun, 130021 China; 2 Department of Intensive Care Unit, First Hospital of Jilin University, Changchun, 130021 China; 3 Institute of Theoretical Chemistry, Jilin University, Changchun, 130021 China; 4 Department of Pathogenobiology, College of Basic Medical Sciences, Jilin University, Changchun, 130021 China

**Keywords:** Blood-brain barrier, Central nervous system infections, Infection

## Abstract

*Toxoplasma gondii*, a widespread obligate intracellular parasite, can infect almost all warm-blooded animals, including humans. The cellular barrier of the central nervous system (CNS) is generally able to protect the brain parenchyma from infectious damage. However, *T. gondii* typically causes latent brain infections in humans and other vertebrates. Here, we discuss how *T. gondii* rhoptry proteins (ROPs) affect signaling pathways in host cells and speculate how this might affect the outcome of *Toxoplasma* encephalitis.

## Facts

- In immunocompromised patients or in individuals with an immature immune system, *Toxoplasma* infection may lead to severe clinical manifestations, such as *Toxoplasma* encephalitis (TE), which in more extreme cases may lead to death.
- ROP plays multiple roles in *T. gondii* infection, such as participating in host cell invasion, monitoring immune signals from host cells, and acting as an important virulence factor in *T. gondii*.
- Studying the effect of ROP will offer broad research prospects for the treatment of toxoplasmosis.

## Open questions

- How can ROP be regulated so that ROP can have different functions under different conditions?
- Immune cells are thought to promote the transmission of *T. gondii* in the brain, which may be one of the methods that the parasite uses to reach neurons.Are there other routes involved in the transmission of *T. gondii* in the brain?
- Can drugs be used to treat toxoplasmosis by inhibiting the expression of ROPs?

## Introduction

*Toxoplasma gondii* (*T. gondii*) is a zoonotic parasite that belongs to the phylum Apicomplexa [1]. Although the seropositivity rate has decreased in European countries over the past 20 years [2, 3], the prevalence of *T. gondii* remains very high in African countries [4, 5]. *T. gondii* can infect a host in a variety of ways. When humans and other intermediate hosts are infected via oocysts or tissue cysts from ingested parasites in contaminated food or water, parasites at the sporozoite and oocyst stages can enter the intestinal tract, pass through the blood and lymphatic system, and then gradually spread to various organs and tissues in the body to become tachyzoites [6]. Tachyzoites may transform into bradyzoites and form cysts in response to attack by the host’s immune system. Expectant mothers infected with *T. gondii* during pregnancy may experience vertical transmission of the parasite to the fetus, resulting in fetal malformation or abortion [7]. In addition, *T. gondii* is a central nervous system (CNS) parasite that can cause toxoplasmosis encephalitis (TE) after infection [8]. TE has high incidence and mortality rates [9]. Furthermore, the host may exhibit neurodegenerative diseases, such as Alzheimer’s disease, paralysis, and epilepsy, after *T. gondii* enters the CNS [10].

Throughout an infection, it is important to understand how the parasite reproduces within host cells and avoids host cell defenses. The early stage of invasion by *T. gondii* is achieved through the interaction between *T. gondii* transmembrane (TM) proteins and surface receptors on the host cell in a process that involves three steps: (1) Identifying host cells. *T. gondii* relies on its unique actin-based gliding motility to glide on the surface of the cell membrane by binding between receptors on the surface of the worm to ligands on the cell surface. (2) Forming a “moving junction” (MJ). After the parasite finds a specific location on the host cell surface for invasion, a tight junction (TJ) structure between its apical tip and the host cell membrane forms. Then, relying on the sliding system and related secreted proteins, the worm rapidly internalizes into the host cell. (3) Forming parasitophorus vacuoles (PVs). Once inside the cell, the invaginated host cell membrane wraps around the body of the parasite to form a PV. At the same time, under the action of dense granule (GRA) proteins, the composition of the PV membrane changes, preventing it from fusing with the host cell. In addition, PV membranes can be connected to parasites through the intravacuolar network (IVN) of host-derived lipid nanotubes. Parasites use secreted effectors to maintain the PV and IVN membranes, recruit nutrients into vacuoles, and evade immune recognition. Ultimately, *T. gondii* undergoes further development and proliferation within PVs.

*Toxoplasma* proliferates by endodyogeny in any nucleated cell type. This process mainly involves replication of the Golgi apparatus in late G1 phase, the replication of centrosomes in S early phase, and budding in S late phase. During this period, the division of the nucleus, endoplasmic reticulum (ER), and mitochondria also occurs. When the offspring parasite matures, the maternal membrane covers the offspring surface. The following parasite division cycle will continue until host cell resources are depleted, ultimately leading to the active exit from and destruction of the infected host cell.

During the whole invasion process, secretory organelles, including micronemes (MICs), dense granules, rhoptries and others, are all involved.

### MICs

Microndosome proteins are also secreted in the free state, but their abundance may increase during invasion; microndosome proteins contain an adhesin domain, suggesting that microndosome proteins may participate in adhesion to host cells. Furthermore, microphytene proteins were found to operate in the form of complexes, such as the TgMIC1/4/6 and TgMIC2/M2AP complexes.

The TgMIC1/4/6 complex is the first and most extensively identified microfilament complex and has been extensively studied in *T. gondii*. This protein complex consists of the soluble adhesion proteins TgMIC1 and TgMIC4 and the TM protein TgMIC6. MIC1 and MIC4 are exposed to the tachyzoite surface and bind host cell surface receptors, and MIC6 binds the complex to the parasite surface. The interacting domains include the following: the second and third EGF-like domains of MIC6, which interact with the C-terminal galectin-like domain of MIC 1; the N-terminal microlinear adhesion repeat (MAR) domain of MIC1; and the first two domains, which bind the Apple domain of MIC4. Together, these proteins promote tachyzoite adhesion and subsequent host cell invasion while also participating in pathogenesis and immune escape.

MIC2 is a TM member of the complex whose extracellular domain consists of the whole I domain followed by five thrombospondin-like repeats (TSRs) and a degenerate sixth TSR close to the TM domain. M2AP is a soluble protein with an N-terminal propeptide, a central beta domain, and a predicted C-terminal helical domain. The hydrophobic residues on the folded side of the M2AP galectin bind the membrane-proximal sixth platelet-reactive protein type I repeat domain of MIC2. After complex formation, MIC2-M2AP can bind host cell surface receptors, which is essential for insect body movement and rapid invasion.

### Rhoptries

After the micronemes participate in the attachment and penetration of *T. gondii*, rhoptries, which are unique apical secretory organelles shared exclusively by all apicomplexan parasites, are involved in parasite penetration and the avoidance of intracellular killing. Rhoptries also contained homogeneous dense organelles resembling dense granules (101). Rhoptries can be divided on the basis of structure into rhoptry neck proteins (RONs) and ROPs. RONs are mainly involved in forming apicomplexan and are involved in mobile junction formation during *T. gondii* invasion. ROPs mainly play three roles. First, ROPs are involved in invasion of the host cell, as they enhance invasion, vacuole formation and the exchange of materials and information with the host cell. Second, ROPs monitor immune signals from the host cell and avoid host cell killing. Third, some rhoptry proteins (ROPs) are important virulence factors of *T. gondii*. Therefore, rhoptries can be used as an entry point to study drug targets and vaccine antigen candidates. To date, approximately 54 kinds of ROPs have been identified. The role of ROPs in *T. gondii* infection and potential therapeutic strategies are shown in Table 1.

**Table 1:** The main features and functions of some ROPs.

Name	Role of ROPs in *T. gondii* infection and potential therapeutic strategies
ROP1	ROP 1 can reduce parasite sensitivity in mice and inherent immune limitations in humans [1].
ROP2	ROP2 is an important virulence factor secreted by the parasite into the host cells and has been recommended as a vaccine candidate for toxoplasmosis [2].
ROP4	An oral ROP 4 vaccine helps against lethal *T. gondii* infection [3].
ROP5	ROP5 is a major determinant of acute toxicity in mice and an immune-related GTPP inhibitor [4].
ROP7	ROP7 can promote inflammasome hyperactivation in ThP-1-derived macrophages by interacting with NLRP 3 [6].
ROP8	ROP8 is expressed during the early stages of infection and plays a key role in PV formation [7].
ROP9	ROP9 is involved in the early stages of host invasion and contains B-cell epitopes. Moreover, a DNA vaccine encoding the ROP 9 gene has shown some immune protection against acute infection [8].
ROP13	ROP13 DNA-based vaccines can act against *T. gondii* by inducing Th 17-associated cytokine production and has been suggested as potential vaccines to control toxoplasmosis [12].
ROP14	ROP14 is a suitable candidate for routine *T. gondii* examination [13].
ROP16	ROP16 can induce macrophages to adopt an M2 phenotype [14]. The deletion of ROP16 aggravated adverse pregnancy outcomes in mice [15].
ROP17	ROP17 both can promote the transmission of *T. gondii* by hijacking monocyte tissue migration and is a vaccine candidate for toxoplasmosis [16].
ROP18	ROP18 inhibits host innate immunity through cGAS-STING signaling [17]. Additionally, ROP18 controls the intracellular proliferation of *T. gondii* [16].
ROP19	A DNA vaccine encoding ROP19 elicited a significant immune response and provided protection against *Toxoplasma* challenge [18].
ROP21	ROP21 could serve as a DNA vaccine against toxoplasmosis [18].
ROP22	ROP22 has been recommended as a vaccine candidate for toxoplasmosis [19].
ROP29	ROP29 has been recommended as a vaccine candidate for toxoplasmosis [20].
ROP35	Vaccination with ROP35 triggers strong cell-mediated and humoral immunity and partially induces defense mechanisms against *T. gondii* [21].
ROP38	ROP38 is a key manipulator of host gene expression and functions in the transformation of tachyzoites to bradyzoites [22]. In early infection, ROP 38 affects parasite invasion and exit and induces the secretion of IL-18 [23].
ROP39	ROP39 mediates an IRGB10-specific parasitic effect and regulates acute Toxoplasma virulence [24].
ROP54	ROP54 has been recommended as a vaccine candidate for toxoplasmosis [25]. ROP54 modulates *T. gondii* virulence and host GBP2 loading [26].

### Dense granules (GRAs)

GRAs are unique organelles of the Apicomplexan parasite and can exist in multiple locations, for example, within the parasitophorous vacuole membrane (PVM) and on the host cytoplasmic surface as well as within the host cytoplasm and nucleus. The functions of GRAs include modification of the vacuolar membrane, parasite body growth and transcriptional regulation of host cells, and they are immunogenic in the host. At present, a variety of GRA proteins have been reported, among which GRA1 is involved in regulation of the vacuolar membrane network of the worm body. GRA2 is involved in regulating the vacuolar membrane network of *Toxoplasma* after invading host cells and can form multimeric complexes with GRA4 and GRA6 to maintain network structural morphology. GRA3 is secreted in *T. gondii* tachyzoites and contains a signal peptide and a TM region at its N-terminus, causing it to be embedded in the form of oligomers on the vacuolar membrane of parasites. GRA4, similar to GRA1 and GRA2, is secreted into the tachyzoite structure. GRA5 is secreted onto the vacuolar membrane to maintain membrane stability. GRA7 is expressed in *T. gondii* at both the asexual and sexual reproductive stages and is the only membrane-intrinsic protein that connects the worm somatic membrane to the host cell membrane. GRA15, GRA16 and GRA24 are among the few GRAs that can enter the host cytoplasm through the vacuolar membrane. GRA15 can enter the host nucleus and participates in regulating host transcription; studies have shown that GRA16 plays an important role in the pathogenicity of *T. gondii*, and GRA24 independently regulates IL-12 in the NF-κB pathway.

## Structure of the blood‒brain barrier (BBB) in vertebrates

The CNS in vertebrates is protected by many cellular barriers that limit and regulate the ability of certain molecules and cells to enter the brain parenchyma [11]. Three main barriers, the BBB, blood-cerebrospinal fluid barrier, and cerebrospinal fluid-brain barrier, protect neurons from harmful substances transmitted through the bloodstream [12]. Pathogens that enter the CNS must do so via one of these routes. Recent research has not demonstrated the ability of *T. gondii* to invade the brain parenchyma through the cerebrospinal fluid barrier. Therefore, when *T. gondii* invades the CNS, it primarily does so by crossing the BBB [13].

The BBB is a special barrier structure that prevents neurotoxic plasma components, blood cells, and pathogens from entering the brain. Highlighting the relationships between cells related to the BBB, they exist in a functional unit called the neurovascular unit (NVU), which is composed of neurons, microglia, vascular endothelial cells, pericytes, astrocytes and extracellular matrix [14]. In these tissues, the factors that affect the function of the BBB mainly include cellular factors and TJs [15].

## Role of the ROP of *T. gondii* in crossing the BBB during parasite infection

**Fig. 1:**
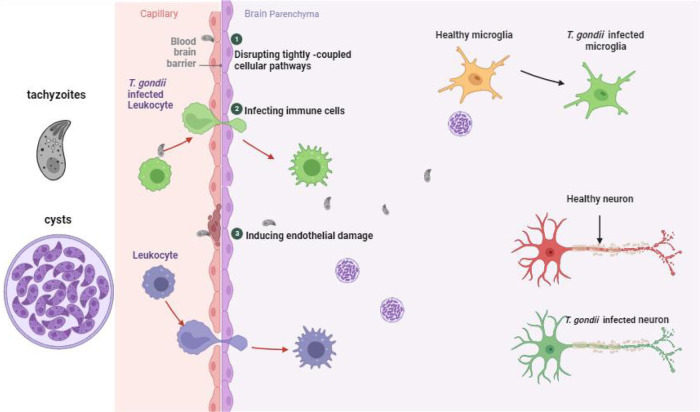
The currently understood mechanisms by which *T.gondii* from the blood circulation penetrates the brain parenchyma. (1) Disrupting intercellular connections. (2) Utilizing the “Trojan horse” mechanism. (3) Inducing endothelial damage to the brain, leading to breakdown of the BBB. Created with BioRender.com.

*T. gondii* is currently known to cross the BBB through the following mechanisms (Fig. 1): (1) by disrupting tightly coupled cellular pathways [16], (2) by infecting immune cells (the “Trojan horse” mechanism) [17], or (3) by inducing endothelial damage to the brain, leading to breakdown of the BBB [18].

### Paracellular pathways that disrupt intercellular connections

TJs are important components that maintain BBB function and are mainly composed of occludin, claudin, connective adhesion molecules and endothelial cell-selective adhesion molecules. TJs are connected to the actin cytoskeleton through the TJ protein zonula occludens (ZO) [19]. The integrity of the BBB is related to dynamic changes in TJ proteins. These dynamics are also the key to the highly selective permeability of the BBB [20]. Ramírez-Flores et al. [21]. showed that proteins present in the excretory/secretory products (ESPs) of *T. gondii* can disrupt the TJs of adjacent cells. After ESP treatment, the expression levels of ZO-1, occludin, and claudin-1 gradually decreased. By analyzing the composition of three kinds of ESPs (ectosomes, exosomes and exosome supernatant fractions), among the detected ROPs, the ROP1, 4, 5, 7, and 8 proteins were found to be present at the highest concentrations among the three components analyzed. Other proteins, such as ROP13, ROP18, and ROP40, were expressed at low levels [21]. However, interestingly, of the few studies of the roles of the ROP1, 4, 5, 7, and 8 proteins in TJs, most of them have focused on their roles in vaccines.

The ROP1 protein is a soluble secreted antigen that plays an important role in the invasion of host cells by *T. gondii* [22]. ROP1 is conserved in the highly virulent RH strain of *T. gondii* and promotes parasite growth in mouse and human macrophages [23]. ROP1 interacts with the host cell protein C1QBP, a newly recognized innate immune signal regulator. Therefore, ROP1 has been suggested as an important innate immune system regulator in both mice and humans [23]. However, there has been relatively little research on the role of ROP1 in TJs.

The roles of ROP4 include the regulation of host cell invasion, parasitic proliferation, and parasitic virulence regulation [24]. At present, research on ROP4 has mainly focused on various vaccines, including oral recombinant vaccines [25], virus-like particle (VLP) vaccines [26], recombinant baculovirus vaccines [27], and others. Research has shown that compared to a combination of two vaccines (ROP4VLP + ROP13VLP), the single ROP (4 + 13) VLP vaccine better promoted the expression of IgG, IgG1, IgG2a, and IgA in mouse serum. Compared to mice immunized with other vaccines (ROP4 VLP, ROP13 VLP, and ROP4 VLP + ROP13 VLP), mice immunized with ROP (4 + 13) VLP exhibited higher IgG and IgA antibody responses in fecal, urine, intestinal, and vaginal samples after infection via oral attack [26].

The ROP7 and ROP4 proteins are 71% identical, including the amino acids responsible for all of the characteristics required for kinase activity. ROP7-knockout (KO) parasites exhibited a reduced inflammatory response by THP-1-derived macrophages, but the ROP7 gene has little effect on the reproductive speed of *T. gondii*. Other research shows that ROP7 is not a virulence factor, and the absence of ROP7 does not affect the invasion, proliferation or excretion of parasites [28].

Recent studies have shown that ROP5 is a major virulence factor, as ROP5 is necessary to cause fatal disease in mice [29]. Genetic screening showed that a polymorphism in ROP5 underlies the differences in virulence between *T. gondii* strains in infected laboratory mice [30]. The study found that deleting type II ROP5 essentially eliminated chronic infections and significantly reduced the virulence mortality rate by >100-fold, indicating that type II ROP5 is a virulence factor [31]. Moreover, a recent study analyzed the interaction between the parasite virulence proteins ROP5B and ROP39. In addition, the direct binding of ROP5B to Irga6 and the inhibition of the IRG resistance system by enhancing ROP18 kinase activity were found to be related to ROP39. Functional analysis of ROP39 showed that it is located on the PVM. Although separate RHΔROP18 *T. gondii* type I exhibited a relatively small impact on virulence in vivo, compared with the RHΔROP18 strain, the toxicity of the double deletion mutant RHΔROP18/ROP39 (double KO *T. gondii* strain) was significantly decreased [32].

ROP8, which belongs to the ROP2 protein family, is the most abundant protein in the ectosome and exosome [21]. Although only a few studies have used ROP8 as a diagnostic marker for toxoplasmosis [33], studies have used ROP8 as a potential candidate vaccine [34]. Research has shown that vaccination with a ROP8 DNA vaccine produced significant humoral and cell-mediated immune responses and conferred significant protective effects to mice against deadly parasitic attack [35]. Mice immunized with ROP8 pVAX-1 DNA had a 100% survival rate before 9 days postinfection (dpi), while all control mice died [36]. However, the mechanism by which ROP8 affects TJs is not yet clear.

### Trojan horse mechanism

The “Trojan horse” mechanism refers to the use of infected cells as carriers to help pathogens spread and cross biological barriers without being detected [37, 38]. Studies have shown that various pathogens can cross biological barriers using the “Trojan horse” mechanism. For example, *Listeria monocytogenes* (Lm) causes bacterial encephalitis by crossing the BBB using the “Trojan horse” mechanism [39], and flaviviruses spread into the nervous system using the “Trojan horse” mechanism [40]. Leukocytes play an important role in the “Trojan horse” mechanism.

The migratory function of leukocytes makes them a suitable vector for *T. gondii* [41]. Dendritic cells (DCs) in *T. gondii*-infected leukocytes, but not in macrophages, show enhanced migration after parasitism by *T. gondii* associated with the GABA/L-VDCC/Cav1.3 motogenic signaling axis [42]. However, when endothelial cells come into contact with DCs, DCs adhere to the endothelium and move in an integrin-dependent manner [43]. This causes a significant reduction in the motility of DCs. Furthermore, CD11b^+^ cells were shown to play a very important role in this process [44]. CD11b^+^/CD11c^+^ cells exhibited the highest infection rate among all peripheral blood mononuclear cells (PBMCs), whereas the majority of the infected cells that migrated across the BBB were CD11b^+^/CD11c^+^ cells. Two types of ROPs play an important role in this process.

TgWIP, a newly identified secreted ROPs that is not harmful to host cells during parasitic invasion, plays an important role in parasite transmission from the infection site to the brain by inducing the excessive movement of DCs [45]. The KO of TgWIP affected the transmission of *T. gondii* to distal organs. In addition, this protein can regulate actin dynamics by interacting with the WAVE regulatory complex (WRC) and SHP2 phosphatase in host cells [46, 47]. The WRC promotes actin polymerization and the formation of pseudopodia, which are closely related to cytoskeletal regulation [48]. SHP2 is a key regulatory factor for the integrity of the blood‒testis barrier (BTB) and BBB [49, 50]. Because TgWIP can affect the morphology of DCs and promote their adhesion to the extracellular matrix, TgWIP enhances the movement and migration of parasitic DCs and may facilitate a “Trojan horse” mechanism for parasites to spread through the host.

TgROP17, which belongs to the ROP2 family, is a serine/threonine kinase [51]. Previous studies have shown that ROP17 can control acute toxicity in mice by synergistically forming complexes with *T. gondii* ROP5 and ROP18 [52]. However, the functions of ROP17 in different genotypes of *T. gondii* differ [53]. Research has shown that ROP17 in genotype I *T. gondii* contributes to cancer immune regulation [54], possibly due to its ability to inhibit the innate immune response of host cells and promote their survival [55]. Deletion of ROP17 in the type II *T. gondii* Pru strain was shown to almost completely eliminate the formation of cysts in the brain [56]. This is mainly because ROP17 promoted the growth of *T. gondii* type II in vitro and, in synergy with ROP18, protected parasitic vacuoles from blocking the host’s immune-related GTPase (IRG)-mediated immune responses [54].

Recent research shows that the main roles of ROP17 in the BBB are to activate Rho/ROCK-dependent processes, to promote monocyte migration in tissues, and to help monocytes quickly reach the BBB [57], rather than helping parasites penetrate the BBB through exosmosis. The migration of parasitic monocytes and macrophages requires host Rho/ROCK signaling and secretion of the parasitic kinase ROP17, which is also necessary for effective transmission in vivo [57]. During the infection of monocytes with *T. gondii*, Rho upregulates interstitial migration to promote the rapid transmission of infected cells within tissues and systemic parasitic transmission [58]. Conversely, the inability of infected monocytes to effectively complete integrin-mediated adhesion or endothelial migration processes indicates that they are not suitable to act as “Trojan horses” to transmit parasites in the BBB. Therefore, the activation of ROP17-dependent interstitial tissue migration in infected monocytes can lead to the inhibition of integrin-dependent processes in endothelial cells.

### Induced disruption of the BBB by the induction of brain endothelial damage

Previous studies have shown that *T. gondii* can cross biological barriers using paracellular migration or by infecting host cells and then crossing the BBB. Recent studies show that parasites can replicate in endothelial cells before they invade the CNS [59, 60]. In these parasites, the ROPs toxofilin and Rab11 also play a very important role in cell invasion.

Toxofilin is a 27 kDa protein that can regulate actin dynamics through monomeric actin. Through protein sequence analysis, toxofilin was found to contain an N-terminal signaling sequence for secretion, mainly present in rhoptries, which plays a crucial role in host invasion [61]. Research shows that toxofilin promotes correct vacuole folding by targeting the host cortical actin cytoskeleton, thus facilitating the invasion process [62].

*T. gondii* Rab11 proteins (Rab11A, Rab11B, and Rab11C/Rab25) are present in different cell compartments, such as the trans-Golgi network (TGN), posterior Golgi vesicles, and circulating nuclear endosomes around the centriole (RE) [63]. Rab11A is involved in phagocytosis, synaptic function, and cell migration. Additionally, Rab11A regulates not only eukaryotic cell exocytosis but also the movement of extracellular parasites and adhesion to host cells [64].

Notably, secretion of the microneme protein MIC2 is altered in Rab11A-deficient parasites, which also exhibit severe morphological defects [65]. Shield-1 was used to treat extracellular Rab11A-WT and KO (Rab11A-DN) parasites for 2 h, and their ability to adhere to host cells was then monitored. Compared to the Rab11A-WT parasite, the Rab11A-DN tachyzoites were severely damaged in terms of their ability to adhere to the monolayer surface of human foreskin fibroblasts (HFFs). In addition, parasites that successfully adhered exhibited strong motor deficits. Importantly, compared to that of the Rab11A-WT parasite, the morphology of the adherent motile Rab11A-DN parasite became wider and shorter, and these parasites were without the typical arc. These defects of Rab11A-DN parasites in terms of host cell adhesion and movement may be due to impaired transmission of MIC2 to the plasma membrane (PM) [65]. This suggests that Rab11 may also play a crucial role in cell invasion.

## The role of *T. gondii* ROPs in the infected brain parenchyma

Previous studies have shown that *T. gondii* enters the brain through direct infection and lysis of BBB endothelial cells or through the “Trojan horse” mechanism, but little is known about the transmission of parasites within the brain. Although various cells can infect parasites, current research generally supports the concept that carrier immune cells can mediate transport [66].

According to recent research by Schneider et al. [67], although extracellular *T. gondii* generally moves faster than cells infected with parasites in vitro, the migration of extracellular *T. gondii* in the brain parenchyma is slower than that of cells infected with parasites. The movement of cells infected with *T. gondii* can be divided into two types. One type of cell movement, which was seen in microglia in vitro, may involve excessive migration after infection with *T. gondii* [68], but movement remained also static in vivo [67]. The movement of other types of cells, such as peripheral infiltrating immune CD45^+^ cells, can transport *T. gondii* tachyzoites through the brain and increase their transmission speed; these parasite-carrying CD45^+^ cells still have a slower average transmission rate in the brain than uninfected CD45^+^ cells [68, 69]. Because microglia play a protective role in maintaining the health of myelin, the presence of fewer microglia will lead to the disintegration of myelin and thus affect cognitive function [70]; this study also suggests that immune cells play dual roles in neuroprotection and promoting the spread of parasites in the brain.

Although astrocytes, microglia and neurons are easily infected in vitro, Cabral et al. [71]. found that parasites tend to be located in neurons. It was not previously clear how *T. gondii* reached neurons, but because immune cells have been suggested to promote the spread of parasites in the brain, this may be one of the methods by which parasites reach neurons. The infection process may involve the initial infection of immune cells followed by transmission to neurons, causing neuronal infection. ROP18 may play a role throughout this process.

In response to various brain injuries, microglia are activated and polarize to form M1-type microglia (proinflammatory) or M2-type microglia (anti-inflammatory) [72, 73]. The immunoregulatory molecules produced by M1 and M2 microglia, which include various inflammatory factors and chemokines, are closely related to brain injury and brain repair, respectively [74–76]. Studies have shown that *T. gondii* ROP18 interacts with purinergic P2X1, a purinergic receptor in SF268 human nerve cells, and suppresses ATP-induced apoptosis via the mitochondrial pathway [77]. This helps to clarify the potential mechanism underlying neuroinflammation mediated by activated microglia. In addition, because the ER-related protein RTN1-C is the substrate for phosphorylation by *T. gondii* ROP18, when ROP18 phosphorylates RTN1-C, this promotes neuronal apoptosis through inducing ER stress. Studies have shown that ROP18 can phosphorylate the serine residues at sites 7 and 134 and threonine residues at sites 4, 8 and 118 in RTN1-C. Phosphorylated RTN1-C can inhibit histone deacetylase (HDAC) activity and promote the acetylation of glucose-regulated protein 78 (GRP78), and highly acetylated GRP78 can upregulate the unfolded protein response, leading to ER stress-related apoptosis [78]. This may be an important mechanism of neuronal apoptosis during the process of toxoplasmosis encephalitis (TE).

ROP 18 is a *T. gondii* kinase that phosphorylates IRGA6, thereby preventing worm body death caused by the aggregation of IRGA6 on the PV. ROP18 can form a complex with ROP5, and this complex can bind the GRA protein GRA7. Furthermore, the indirect interaction between an effector immunity-related GTPase (IRG), IRGA6, is mediated by ROP5. Thus, ROP18 should regulate IRGA6 by binding ROP5, and the presence of ROP5 in this complex causes IRGA6 to be phosphorylated after binding by G7 [79]. In neural cells, worm strains expressing high levels of ROP18 did not contain IRGA6 on the PV regardless of IFN-γ stimulation. Other insect strains stimulated by IFN-γ showed the activation of IRGs, which eventually removed intracellular parasites in vitro and in vivo [80].

In ROP16 plays an additional very important role. In a vesicular stress model and in mouse primary neuronal cell culture (PNC), the absence of ROP16 did not affect type II parasite encystation but significantly reduced type III parasite encystation. Furthermore, the phosphorylation and activation of the host cell transcription factor STAT6 by the parasitic kinase ROP16 were found to be necessary for encystation in *T. gondii* type III strains. Research has shown that compared to WT type III and ROP16 type III parasites, IIIΔ ROP16 and ROP16 type II parasites showed a significant reduction in cysts (30-50%) at 4-8 dpi [81]. Meanwhile, the loss of ROP 16 in type III parasites caused a significantly reduction in parasite numbers and enhanced parasite-specific T-cell responses, suggesting that ROP16 is a virulence factor [82].

Currently, the size and number of cysts in the brain are weighted indicators used to measure the quality of *T. gondii* vaccines. Therefore, vaccines prepared with various ROPs have shown various impacts on the size and quantity of cysts in the brain [1, 83]. For example, ROP4 and ROP13 VLP vaccines play a specific role in inducing intracranial antibody responses [26]. Furthermore, intracranial IgG and IgA responses in groups treated with ROP4 VLP and ROP13 VLP vaccines were significantly stronger than those in the infected group. Meanwhile, the IFN-γ and IL-6 levels in the ROP4 VLP treatment group were significantly lower than those in the infected group. In addition, cyst sizes in the ROP4 and ROP13 VLP treatment groups were significantly smaller than those in the infected group. Furthermore, the number of cysts in mice immunized with the vaccine were also significantly decreased. The efficacy of the vaccine was evaluated by the attack of infected and immunized mice with *T. gondii* ME49. Immunization with VLPs could prevent severe weight loss and ensured 100% survival, while nonimmunized mice gradually lost body weight and eventually died.

## Conclusion

Encephalitis is the most important manifestation of toxoplasmosis in immunosuppressed patients, as it causes the most severe damage to patients. Infection can occur in any organ. Patients may experience headaches, disorientation, drowsiness, hemiplegia, altered reflexes, and convulsions, with many falling into a coma. Most lesions in the brain are necrotic, especially in the thalamus. Although recent progress has contributed to a better understanding of the role of certain *T. gondii* ROPs in the outcome of TE, many issues still need to be addressed. For example, there are many ways to transport parasites through the BBB, but more work needs to be done to elucidate the role of each mechanism. In addition, it should be noted that the same ROP can have different functions under different conditions. Because some ROPs have been mainly investigated in terms of their extracellular effects, their functions have not yet been fully explored. However, ROP antigens are strong vaccine candidates, and future research on ROPs might therefore be a major direction in addressing *T. gondii* infection.
